# Pharmacy practice in hospital settings in GCC countries: Pharmacists’ medication therapy monitoring activities

**DOI:** 10.1016/j.jsps.2024.101952

**Published:** 2024-01-04

**Authors:** Ahmed H. Al-jedai, Ahmed Y. Mayet, Fowad Khurshid, Mohammed S. Alsultan

**Affiliations:** a Therapeutic Affairs, Ministry of Health, Riyadh, Saudi Arabia; b Colleges of Medicine and Pharmacy, Al-Faisal University, Riyadh, Saudi Arabia; c Department of Clinical Pharmacy, College of Pharmacy, King Saud University, P.O. Box: 2457, Riyadh 11451, Saudi Arabia; d Department of Pharmacy, Institute of Biomedical Education and Research, Mangalayatan University, Aligarh 202145, India

**Keywords:** Medication therapy, Therapeutic drug monitoring, Drug therapy management, Adverse drug events

## Abstract

**Purpose:**

Our study aims to provide an overview of medication therapy monitoring practices carried out by pharmacists in hospitals across the Gulf Cooperation Council (GCC) countries.

**Methods:**

This is a cross-sectional questionnaire-based study of hospitals located in the GCC. Questions were adopted from the American Society of Health-System Pharmacists (ASHP) national survey. Frequency analyses were used to examine the number and percentages of specific responses to the survey questions.

**Results:**

A total of 64 hospitals participated in this survey, reflecting an overall response rate of 52.0%. Almost half of participating hospitals (48.4%) were from Saudi Arabia. Among the 64 participating hospitals, 54.7% monitored their patients daily, 40.6% assigned pharmacists to patient care units for at least eight hours per day, and 42.2% held pharmacists accountable for medication-related outcomes. Moreover, the criteria used to identify patients requiring monitoring, 35.9% relied on the list of high-risk medications, 26.5% relied on specific medical services, 21.9% relied on directions from the hospital committee, and 17.2% relied on lab abnormalities. The most frequently utilized method for monitoring adverse drug events (ADEs) was through notifications from nurses or physicians, observed in 60.9% of participating hospitals.

**Conclusion:**

The survey emphasizes the need for hospitals in the GCC to promote increased pharmacist accountability for medication-related outcomes, explore technological solutions to enhance monitoring efficiency and extend the presence of pharmacists in patient care units beyond the current level.

## 1 Introduction

The role of pharmacists in hospital settings, especially in ensuring optimal medication therapy during inpatient admissions, has been increasingly recognized as vital to patient care. As Schmidt et al. (2020), have noted, pharmacists are instrumental in monitoring patients' responses to specific medications, a key component in ensuring safe, effective, patient-centered care (Schmidt et al., 2020). This role extends beyond the only dispensation of drugs, involving a deep engagement with therapeutic drug monitoring services. Studies by Falch and Alves (2021), Alhameed et al. (2019), Hussain et al. (2021), and Firman et al. (2022), have consistently shown positive outcomes from pharmacist-managed services, including shorter treatment durations and a reduction in adverse drug reactions (Alhameed et al., 2019, Falch and Alves, 2021, Hussain et al., 2021, Firman et al., 2022). The significance of pharmacists in drug therapy management is further underscored in the works of Wang et al. (2021), and Meng et al. (2023), (Wang et al., 2021, Meng et al., 2023).

Pharmacists in the Gulf Cooperation Council (GCC) region is poised to face significant challenges in medication therapy monitoring arising from various barriers. Healthcare teams may not fully utilize the expertise of pharmacists, affecting patient outcomes. Limited access to patient records and laboratory data impedes effective monitoring. The lack of uniform protocols can hinder collaboration with other healthcare professionals, and variability in education and training across the region affects pharmacists' capabilities. Furthermore, regulatory differences among GCC countries and uneven technological integration, such as in electronic health records, present additional challenges. Addressing these issues is vital for enhancing pharmacy practices in the GCC.

Our study, based on the ASHP questionnaire, investigates how pharmacists in the GCC region monitor patients in hospitals. We focus on several key areas: how healthcare teams use pharmacists' skills, whether pharmacists have easy access to complete patient records and lab results, the effectiveness of safety measures in medication use, how they identify adverse drug events (ADEs), the role of multidisciplinary committees, the implementation of projects to improve quality, and the use of technologies like electronic health records. Understanding and addressing these challenges is essential for appreciating the role of pharmacists in the GCC and improving pharmacy practices.

Building on our prior work, “Hospital Pharmacy Practice in Saudi Arabia: Drug Monitoring and Patient Education in the Riyadh Region” from 2013 (Alsultan et al., 2013), this study broadens our research horizon. The previous study, while insightful, was confined to Riyadh and highlighted considerable variations in pharmacists' involvement in medication therapy monitoring. Our current research extends this scope to include the entire GCC region, offering a more holistic understanding. This expansion enables us to gather insights that exceed the Saudi Arabian context and formulate strategies to improve pharmacy practices throughout the region.

This publication represents the third installment in a series, following the works of Al-Jedai et al. (2021), and Mayet et al. (2023), (Al-jedai et al., 2021, Mayet et al., 2023). It delves into the roles of pharmacists in medication therapy monitoring as part of the medication use process in hospitals across the GCC countries.

## 2 Methods

### 2.1 Study design

This cross-sectional questionnaire-based study comprised 64 hospitals from five countries in the GCC, including Saudi Arabia, Kuwait, the United Arab Emirates (UAE), Oman, and Bahrain. The questionnaire was adapted from the American Society of Health - System Pharmacists (ASHP) national survey on pharmacy practice (Pedersen et al., 2015, Schneider et al., 2018). Furthermore, this questionnaire was reviewed by the research team in our group, providing comprehensive feedback on adding or removing questions as necessary. Additionally, discussions with the pharmacy directors from different hospitals were conducted to enhance the clarity and relevance of the questions used in the questionnaire. The online platform “Google Forms” was used to create the survey due to its user-friendly nature and compatibility with various web browsers (Rayhan et al., 2013). The effectiveness and accessibility of this platform were comparable to the ASHP survey method (Pedersen et al., 2015, Schneider et al., 2018, Pedersen et al., 2021).

The survey consists of three parts. The first part focuses on medication therapy and therapeutic drug monitoring activities, extracting information about the characteristics of patients who require monitoring, the criteria for selecting patients, and the data collection process for monitoring. The second part focuses on drug therapy management activities, extracting information about the presence of pharmacists assigned to patient care or specialty services and those responsible for drug therapy management. The third part focuses on monitoring adverse drug events (ADEs), extracting information about the identified ADEs and the methods used for their identification.

### 2.2 Data collection and statistical analysis

Data collection was carried out using a convenience sampling technique. This non-probability sampling method involved selecting study subjects based on specific criteria such as availability, willingness to participate, accessibility, and proximity to the researchers (Martínez-Mesa et al., 2016). We obtained a list of hospitals from the Ministry of Health in the selected GCC countries to identify potential participants. Subsequently, we sent a secure invitation link along with a survey questionnaire directly to the pharmacy director of each hospital. The responses to the questionnaire were collected from November 2019 to April 2020, and three follow-up attempts were made with non-responding participants within the study period to declare non-responders.

SPSS (version 26.0; IBM Corp., Armonk, NY) was used to run the whole data analysis. Frequency analysis was used to examine the number and percentages of specific responses to the survey questions and hospital characteristics such as country, type of hospital, location, and accreditation. For multiple-response questions, percentages were calculated based on each hospital's total responses.

## 3 Results

### 3.1 Baseline characteristics of participating hospitals

The survey was sent to 123 hospital pharmacy directors. However, only 64 of them responded to the survey. The overall response rate was 52.0 %. Table 1 elucidates the baseline characteristics of the 64 hospitals. 14 hospitals (21.9 %) have between 100 and 199 staffed beds. The majority of participating hospitals (48.4 %) were from Saudi Arabia. 25 hospitals (39.1 %) were general hospitals, while 16 hospitals (25.0 %) were specialized. Furthermore, most of the hospitals (73.4 %) were accredited by any of the following organizations: Joint Commission International (JCI), The Saudi Central Board of Accreditation for Healthcare Institutions (CBAHI), and The Canadian Accreditation Body (CAB).

**Table 1:** Characteristics of the 64 participating hospitals.

**Characteristics**	
**Number of Staffed beds**	
<50	7 (10.9 %)
50 − 99	8 (12.5 %)
100 − 199	14 (21.9 %)
200 − 299	6 (9.4 %)
300 − 399	11 (17.2 %)
400 − 499	6 (9.4 %)
500 − 599	5 (7.8 %)
≥ 600	7 (10.9 %)
**Country**	
Saudi Arabia	31 (48.4 %)
Kuwait	21 (32.8 %)
UAE	6 (9.4 %)
Oman	5 (7.8 %)
Bahrain	1 (1.6 %0
**Type of hospital**	
General	25 (39.1 %)
Academic/Teaching	4 (6.3 %)
Secondary care	7 (10.9 %)
Tertiary care	12 (18.7 %)
Specialized	16 (25.0 %)
**Ownership**	
Government hospital	64 (100.0 %)
**Accreditation**	
Accredited*	47 (73.4 %)
Non-accredited	17 (26.6 %)

*Obtained from any of these organizations: JCI, CBAHI, and CAB.

### 3.2 Medication therapy and therapeutic drug monitoring (TDM) activities

Table 2 elucidates the characteristics of pharmacists’ activities in monitoring medication therapy and therapeutic drugs. Approximately half of participating hospitals (54.7 %) had over 75 % of their patients being monitored by hospital pharmacists. Among hospitals whose pharmacists monitor their patients daily, eight hospitals (12.5 %) monitored all their patients, 12 hospitals (18.8 %) used an informal process (e.g., individual pharmacist selection), three hospitals (4.7 %) used formalized paper-based screening, and seven hospitals (10.9 %) used computerized data mining for identifying patients who require monitoring. With regards to the criteria used to identify patients requiring monitoring, 23 hospitals (35.9 %) relied on the list of medications (e.g., high-risk medications), 17 hospitals (26.6 %) relied on the medical service utilized by the patient, 14 hospitals (21.9 %) relied on directions from the hospital committee, and 11 hospitals (17.2 %) relied on the lab abnormalities. Moreover, in 32 hospitals (50 %), physicians, not pharmacists, were responsible for monitoring patients. With regards to the data collection process utilized for pharmacists’ monitoring, 42 hospitals (65.6 %) used manual processes, 24 hospitals (37.5 %) used data mining functionality within hospital electronic health records (EHR), and seven hospitals (10.9 %) used clinical surveillance software (e.g., TheraDoc, Sentri7, and MedMined).

**Table 2:** Characteristics of pharmacists’ activities in monitoring medication therapy and therapeutic drugs.

**Characteristics**	
**Percentages of patients monitored daily by pharmacists**	
<26 %	15 (23.4 %)
26 − 50 %	10 (15.6 %)
51 − 75 %	4 (6.3 %)
>75 %	35 (54.7 %)
**Methods for identifying patients requiring monitoring by pharmacists**	
All patients are monitored daily	8 (12.5 %)
Informal process	12 (18.8 %)
Formalized paper-based screening	3 (4.7 %)
Computerized data mining	7 (10.9 %)
In our hospital, pharmacists rarely monitor	34 (53.1 %)
Criteria used to identify patients requiring monitoring*	
Formalized list of medications (e.g., high-risk medication)	23 (35.9 %)
Specific medical or surgical services (or physical location such as ICU)	17 (26.6 %)
Disease state	5 (7.8 %)
Abnormal lab value changes requiring dosage adjustment	11 (17.2 %)
High-cost medications (e.g., IVIG, Factor VII)	6 (9.4 %)
Informal process (e.g., individual pharmacist selection)	4 (6.3 %)
As directed by hospital committee (e.g., P&T identifying targeted DUE)	14 (21.9 %)
Pharmacists do not monitor patients; physicians are primarily responsible for it	32 (50.0 %)
Data collection for pharmacist monitoring*	
Manual processes	42 (65.6 %)
Clinical surveillance software (e.g., TheraDoc, Sentri7, MedMined)	7 (10.9 %)
Data mining functionality within hospital electronic health records (EHR)	24 (37.5 %)

*Multiple responses were selected - percentages were calculated based on the total number of responses for each hospital.

### 3.3 Drug therapy management activities

Table 3 elucidates the characteristics of pharmacists’ activities in drug therapy management. In 22 hospitals (34.4 %), pharmacists were authorized to monitor medications’ levels (e.g., vancomycin and aminoglycoside) or their surrogate markers (e.g., the international normalized ratio (INR)). In 13 hospitals (20.3 %), pharmacists were authorized to adjust medications’ doses. Additionally, in 10 hospitals (15.6 %), pharmacists were authorized to order measurements of medications’ serum concentrations as per established protocol. Among the 64 hospitals included, 26 (40.6 %) assigned pharmacists to patient care units for at least eight hours per day, and 27 (42.2 %) held pharmacists accountable for patients' medication-related outcomes under their care. The areas where pharmacists were most frequently assigned for at least 8 h per day, five days per week, were medical-surgical, neonatal, and infectious disease units.

**Table 3:** Characteristics of pharmacists’ activities in drug therapy management.

**Characteristics**	
**Therapeutic drug monitoring**	
Monitor medication levels	22 (34.4 %)
Authorized to order serum medication level measurement	10 (15.6 %)
Authorized to adjust doses for a routinely monitored medication	13 (20.3 %)
**Pharmacists routinely assigned to patient care units for at least 8 h per day**	
Yes	26 (40.6 %)
No	38 (59.4 %)
**Pharmacists held accountable for medication-related outcomes of patients under their care**	
Yes	27 (42.2 %)
No	37 (57.8 %)
Hospitals with pharmacists assigned to area for ≥ 8 hr/day*	
Inpatient medical-surgical	12 (18.8 %)
Critical care	11 (17.2 %)
Cardiology	11 (17.2 %)
Oncology	7 (10.9 %)
Organ transplant	5 (7.8 %)
Neonatal	12 (18.8 %)
Obstetrics / Gynaecology	10 (15.6 %)
Operating Room / Preoperative Areas	10 (15.6 %)
Emergency Department	11 (17.2 %)
Specialty services: anticoagulation education	9 (14.1 %)
Specialty services: infectious disease	12 (18.8 %)
Specialty services: nutrition support	10 (15.6 %)
Specialty services: pain and palliative care	8 (12.5 %)

*Multiple responses were selected - percentages were calculated based on the total number of responses for each hospital.

### 3.4 Monitoring adverse drug events (ADEs)

Table 4 elucidates the characteristics of pharmacists’ activities in monitoring ADEs. Forty-eight hospitals (75.0 %) established a multidisciplinary committee responsible for the review, analysis, education, policy development, and corrective actions related to the ADEs. The multidisciplinary committee consisted of physicians, pharmacists, and nurses. With regards to quality improvement, 34 hospitals (53.1 %) utilized systematic quality improvement processes (e.g., Lean and Six Sigma), 37 hospitals (57.9 %) performed at least one retrospective root cause analysis (RCA), and 22 hospitals (34.4 %) performed at least one Failure Modes and Effects Analysis (FMEA) in the previous year to enhance medication safety. With regards to methods of identifying ADEs, 39 hospitals (60.9 %) relied on notifications from physicians or nurses in charge, 28 hospitals (43.8 %) relied on interactions with patients, 23 hospitals (35.9 %) relied on TDM activities, 20 hospitals (31.3 %) relied on routine review of lab values, and 20 hospitals (31.3 %) relied on ADE incident report system.

**Table 4:** Characteristics of pharmacists’ activities in monitoring adverse drug events (ADEs).

**Characteristics**	
**Establishment of multidisciplinary committee**	
Yes	48 (75.0 %)
No	16 (25.0 %)
**Quality improvement**	
Process to improve medication use safety	34 (53.1 %)
Root cause analysis (RCA)	37 (57.9 %)
Failure Modes and Effects Analysis (FMEA)	22 (34.4 %)
Methods to identify ADEs*	
Routine review of lab values	20 (31.3 %)
Therapeutic drug monitoring	23 (35.9 %)
Pharmacists round with physicians to assess ADEs	16 (25.0 %)
Pharmacists round independent of physicians	16 (25.0 %)
Adverse drug events hotline	7 (10.9 %)
Medical record E-codes	2 (3.1 %)
Alerting orders (e.g., stat K + level)	5 (7.8 %)
Notification from nurse or physician	39 (60.9 %)
Through patient counselling / interaction	28 (43.8 %)
ADE incident reporting system	20 (31.3 %)
Chart review	3 (4.7 %)
None of the above methods are used	9 (14.1 %)

*Multiple responses were selected - percentages were calculated based on the total number of responses for each hospital.

## 4 Discussion

This survey, the first of its kind in the Gulf Cooperation Council (GCC) countries, provides crucial baseline information on pharmacy practices within hospitals in the region. Due to limited existing data, the survey results are published stepwise, with this report being the third part in a series. The focus is on monitoring practices in hospitals across GCC countries, contributing valuable descriptive data to the understanding of pharmacy practices in the region.

The survey of 64 GCC hospitals, mainly from Saudi Arabia, details pharmacy practices. Over 50 % have pharmacists monitoring 75 % or more of patients, with about one-third relying on medication lists and another third authorizing pharmacists to monitor medication levels. Notably, three-quarters of the hospitals have multidisciplinary ADE committees, about half of the hospitals allocate pharmacists at least 8 h daily for drug therapy management, and about half of the hospitals hold them accountable for medication outcomes. These results underscore the expanding role and responsibilities of pharmacists in recent times.

The literature highlights the essential role of pharmacists in decision-making and monitoring therapeutic drugs. A retrospective observational study conducted in Egypt in 2018 to document clinical pharmacists' interventions in managing drug-related problems (DRPs) revealed their critical involvement in providing recommendations on medication monitoring, interruptions, and discontinuations. The authors asserted that physicians accepted 90.0 % of the pharmacists' recommendations (Ali et al., 2018). A single-blind controlled study conducted in the United States in 2003 found that the inclusion of pharmacists in healthcare teams resulted in a 78.0 % reduction in preventable adverse drug events (ADEs) compared to teams without pharmacists. Pharmacists recommended 150 documented interventions during the rounding process, and 147 (98.0 %) of these interventions were accepted by the healthcare team. The most common types of interventions included incorporating additional medications and adjusting medication doses (Kucukarslan et al., 2003). A 2023 retrospective study in China concluded that pharmacists, through their involvement in medication therapy management (MTM) services, could identify more medication-related problems (MRPs), promoting rational drug use and ensuring medical safety and effectiveness (Meng et al., 2023). Additionally, a 2023 prospective observational study in India underscored the crucial role of pharmacists in patient safety by detecting various adverse drug reactions (ADRs) (Osoro et al., 2023).

Various studies in literature have proposed different approaches to identify patients who would benefit most from pharmacist-led medication therapy monitoring activities. The criteria for patient identification vary based on the healthcare setting, local protocols, and individual patient needs. For example, a 2018 study in Egypt identified patients requiring monitoring based on factors such as high-risk medications, non-adherence struggles, complex medication regimens, and those actively seeking medication reviews (Ali et al., 2018). This aligns with our study, where 35.9 % of participating hospitals used the list of high-risk medications to identify such patients. Another study in New Zealand (2014) suggested the effectiveness of software-based tools, like the Assessment of Risk Tool (ART), in prioritizing inpatients for adverse drug event (ADE) prevention strategies. ART implementation was associated with increased medication reconciliation, reduced errors, and prevention of potential harm (Falconer et al., 2014).

In our study, the identification of adverse drug events (ADEs) varied among participating hospitals, with 60.9 % relying on notifications from nurses and physicians, 43.8 % on patient counseling, and 31.3 % on routine lab value reviews. These results differ slightly from a 2012 study in Korea, where predominant methods included spontaneous reporting, patient interviews, chart reviews, trigger instruments, and computerized monitoring systems (Yun et al., 2012). However, our findings align with a 2014 Canadian study emphasizing the critical role of monitoring lab values for medication dosage adjustments to enhance patient safety (Jackevicius and Glassman, 2014). A 2018 U.S. study also concurred that pharmacists relied on notifications and patient counseling for ADE identification (Pedersen et al., 2019). Additionally, literature emphasizes multidisciplinary committees and Lean and Six Sigma methodologies to improve healthcare quality (Aung et al., 2022). In our study, 75.0 % of hospitals established such committees, and 53.1 % implemented systematic quality improvement methods, highlighting the importance of pharmacist involvement in decision-making for patient safety.

In contrast to our 2013 questionnaire-based study, this research indicates notable enhancements in pharmacist engagement in patient care and medication therapy monitoring. The earlier study revealed that fewer than 25 % of hospitals had patients monitored by hospital pharmacists (Alsultan et al., 2013). Noticeably, in this recent investigation, over half of the hospitals saw patients being monitored by these professionals. This shift underscores the evolving role of pharmacists over the past few years in GCC countries.

In our earlier study involving 29 hospitals, 39.1 % used the list of medications, medical services, and lab abnormalities each to identify patients requiring monitoring (Alsultan et al., 2013). In this recent study with 64 hospitals, the reliance on these criteria shifted to 35.9 %, 26.6 %, and 17.2 %, respectively. These findings indicate evolving criteria for hospital pharmacists to identify patients for monitoring during admissions, reflecting a growing interest and commitment to enhancing pharmacist involvement in patient care and medication therapy monitoring across GCC countries.

Comparing the results of this study with our previous one highlights the evolving role of pharmacists in GCC hospital settings. The trend shows a heightened responsibility and active engagement of pharmacists in medication therapy management (MTM) and patient education. This increased participation in medication monitoring is poised to enhance patient outcomes and safety in GCC countries.

This study has limitations that need consideration. Firstly, a limited number of hospital directors from the UAE, Bahrain, and Oman participated in the survey, with a predominant focus on Saudi Arabian hospitals. This may restrict the generalizability of findings to the entire GCC region, encouraging the inclusion of a more diverse representation from other member countries for a comprehensive perspective. Additionally, the study heavily relies on descriptive data, limiting the depth of analysis. Utilizing more in-depth qualitative or mixed-methods approaches could provide better insights into pharmacy practices. The reliance on self-reported data can introduce the potential for reporting bias.

## 5 Conclusion

This cross-sectional questionnaire-based study highlights the enhancement of pharmacist-led drug monitoring practices in GCC hospitals. The study also indicates that participating hospitals have embraced increased pharmacist involvement, with dedicated hours for drug therapy management services and accountability for medication-related outcomes. The results signify a positive trend in the expanded role and responsibility of pharmacists in MTM and patient education within GCC hospitals. Future research could explore into assessing the impact of pharmacists' engagement in drug therapy management on patient outcomes across GCC countries.

Future studies should examine pharmacists' varying roles and authority across the Gulf Cooperation Council (GCC) countries and explore how these differences impact their professional practices. Also, it is crucial to investigate how standard practices among pharmacists in different GCC countries influence their effectiveness and efficiency in patient care. Additionally, there is a need to assess the impact of new healthcare policies (if any), advancements in pharmacy education, and the influence of accreditation or training programs on pharmacy practices in the GCC. Understanding these dynamics is critical for comprehending the evolution of pharmacy practices in the region. Furthermore, our current study can offer valuable lessons that could be applicable in different healthcare settings globally, contributing to the global discourse on pharmacy practices.

**Funding**

This research did not receive any specific grant from funding agencies in the public, commercial, or not-for-profit sectors.

## Declaration of competing interest

The authors declare that they have no known competing financial interests or personal relationships that could have appeared to influence the work reported in this paper.

## References

[b0005] Alhameed A., Khansa S., Hasan H., Ismail S., Aseeri M. (2019). Bridging the gap between theory and practice; the active role of inpatient pharmacists in therapeutic drug monitoring. Pharmacy (basel)..

[b0010] Ali M.A.S., Khedr E.M.H., Ahmed F.A.H., Mohamed N.N.E. (2018). Clinical pharmacist interventions in managing drug-related problems in hospitalized patients with neurological diseases. Int. J. Clin. Pharm..

[b0015] Al-jedai A.H., Khurshid F., Mayet A.Y., Al-Omar H.A., Alghanem S.S., Alsultan M.S. (2021). Pharmacy practice in hospital settings in GCC countries: Prescribing and transcribing. Saudi Pharm. J..

[b0020] Alsultan M.S., Mayet A.Y., Khurshid F., Al-jedai A.H. (2013). Hospital pharmacy practice in Saudi Arabia: Drug monitoring and patient education in the Riyadh region. Saudi Pharm .J..

[b0025] Aung A.K., Walker S., Khu Y.L., Tang M.J., Lee J.I., Graudins L.V. (2022). Adverse drug reaction management in hospital settings: review on practice variations, quality indicators and education focus. Eur. J. Clin. Pharmacol..

[b0030] Falch C., Alves G. (2021). Pharmacists' role in older adults' medication regimen complexity: a systematic review. Int. J. Environ. Res. Public Health..

[b0035] Falconer, N., Nand, S., Liow, D., et al., 2014. Development of an electronic patient prioritization tool for clinical pharmacist interventions. Am. J. Health Syst. Pharm. 71, 311-320. 10.2146/ajhp130247. 10.2146/ajhp130247 24481156

[b0040] Firman P., Tan K.-S., Clavarino A., Taing M.-W., Whitfield K. (2022). Pharmacist-managed therapeutic drug monitoring programs within Australian Hospital and Health Services-A National Survey of Current Practice. Pharmacy (basel)..

[b0045] Hussain K., Ikram R., Ambreen G. (2021). Pharmacist-directed vancomycin therapeutic drug monitoring in pediatric patients: a collaborative-practice model. J. Pharm. Policy Pract..

[b0050] Jackevicius C.A., Glassman P. (2014). Laboratory monitoring for pharmaceuticals: familiarity does not breed contempt. J. Gen. Intern. Med..

[b0055] Kucukarslan S.N., Peters M., Mlynarek M., Nafziger D.A. (2003). Pharmacists on rounding teams reduce preventable adverse drug events in hospital general medicine units. Arch. Intern. Med..

[b0060] Martínez-Mesa J., González-Chica D.A., Duquia R.P., Bonamigo R.R., Bastos J.L. (2016). Sampling: how to select participants in my research study?. An. Bras. Dermatol..

[b0065] Mayet A.Y., Khurshid F., Al-Omar H.A., Alghanem S.S., Alsultan M.S., Al-jedai A.H. (2023). Pharmacy practice in hospital settings in GCC countries: Dispensing and administration. Saudi Pharm J..

[b0070] Meng Q., Sun L., Ma Y., Wei Y., Ma X., Yang L.u., Xie Z., Li F., Wang Z., Tao X., Zhen X., Jin R., Gu H. (2023). The impact of pharmacist practice of medication therapy management in ambulatory care: an experience from a comprehensive Chinese hospital. BMC Health Serv Res..

[b0075] Osoro I., Amir M., Vohra M. (2023). Pharmacist Interventions in minimizing drug related problems in diabetes with co-existing hypertension: a five-year overview and ground report from India. Int. J. Public Health..

[b0080] Pedersen, C. A., Schneider, P. J., Ganio, M. C., et al., 2019. ASHP national survey of pharmacy practice in hospital settings: Monitoring and patient education-2018. Am J Health Syst Pharm. 76, 1038-1058. 10.1093/ajhp/zxz099. 10.1093/ajhp/zxz099 31361881

[b0085] Pedersen, C. A., Schneider, P. J., Ganio, M. C., et al., 2021. ASHP national survey of pharmacy practice in hospital settings: Dispensing and administration-2020. Am J Health Syst Pharm. 78, 1074-1093. 10.1093/ajhp/zxab120. 10.1093/ajhp/zxab120 PMC8083667 33754638

[b0090] Pedersen C.A., Schneider P.J., Scheckelhoff D.J. (2015). ASHP national survey of pharmacy practice in hospital settings: Dispensing and administration–2014. Am. J. Health Syst. Pharm..

[b0095] Rayhan R.U., Zheng Y., Uddin E. (2013). Administer and collect medical questionnaires with Google documents: a simple, safe, and free system. Appl. Med. Inform..

[b0100] Schmidt S.J., Wurmbach V.S., Lampert A., Bernard S., Haefeli W.E., Seidling H.M., Thürmann P.A. (2020). Individual factors increasing complexity of drug treatment-a narrative review. Eur. J. Clin. Pharmacol..

[b0105] Schneider P.J., Pedersen C.A., Scheckelhoff D.J. (2018). ASHP national survey of pharmacy practice in hospital settings: Dispensing and administration-2017. Am. J. Health Syst. Pharm..

[b0110] Wang X., Wang S., Yu X., Ma Z., Wang H., Yang J., Liu L. (2021). Impact of pharmacist-led medication therapy management in ambulatory elderly patients with chronic diseases. Br. J. Clin. Pharmacol..

[b0115] Yun I.S., Koo M.J., Park E.H., Kim S.-E., Lee J.-H., Park J.-W., Hong C.-S. (2012). A comparison of active surveillance programs including a spontaneous reporting model for phamacovigilance of adverse drug events in a hospital. Korean J. Intern. Med..

